# Choosing statistical test

**DOI:** 10.4103/0974-7788.72494

**Published:** 2010

**Authors:** Shraddha Parab, Supriya Bhalerao

**Affiliations:** *Department of Clinical Pharmacology, Seth GS Medical College and KEM Hospital, Parel, Mumbai - 400 012, India*; 1 *Department of Clinical Pharmacology, TNMC and BYL Nair Hospital, Mumbai Central, Mumbai - 400 008, India*

## INTRODUCTION

Statistical tests are mathematical tools for analyzing quantitative data generated in a research study. The multitude of statistical tests makes a researcher difficult to remember which statistical test to use in which condition. There are various points which one needs to ponder upon while choosing a statistical test. These include the type of study design (which we discussed in the last issue), number of groups for comparison and type of data (i.e., continuous, dichotomous or categorical).

In the present article, we are going to discuss these points, but before that, let us go through some important concepts in statistics.

There are four main levels of measurement/types of data used in statistics. They have different degrees of usefulness in statistical research. Ratio measurements have both a meaningful zero value and the distances between different measurements defined; they provide the greatest flexibility in statistical methods that can be used for analyzing the data. Interval measurements have meaningful distances between measurements defined, but the zero value is arbitrary (as in the case with longitude and temperature measurements in Celsius or Fahrenheit). Ordinal measurements have imprecise differences between consecutive values, but have a meaningful order to those values. Nominal measurements have no meaningful rank order among values. Because variables conforming only to nominal or ordinal measurements cannot be reasonably measured numerically, sometimes they are grouped together as categorical variables, whereas ratio and interval measurements are grouped together as quantitative or continuous variables due to their numerical nature.

In the study of statistics, we focus on mathematical distributions for the sake of simplicity and relevance to the real world. Understanding these distributions will enable us to visualize the data easier and build models quicker. However, they cannot and do not replace the work of manual data collection and generating the actual data distribution. Distributions show what percentage of the data lies within a certain range. So, given a distribution and a set of values, we can determine the probability that the data will lie within a certain range. The same data may lead to different conclusions if they are interposed on different distributions. So, it is vital in all statistical analysis for data to be put onto the correct distribution.

## *P* VALUE

Assume that there are data collected from two samples and that the means of the two samples are different. In this case, there are two possibilities: the samples really have different means or the other possibility is that the difference that is observed is a coincidence of random sampling. However, there is no way to confirm any of these possibilities.

All one can do is calculate the probabilities (known as “*P*” value in statistics) of observing a difference between sample means in an experiment of the studied sample size. The value of *P* ranges from zero to one. If the *P* value is small, then the difference is quite unlikely to be caused by random sampling, or in other words, the difference between the two samples is real. One has to decide this value in advance, i.e., at which smallest accepted value of *P*, the difference will be considered as real difference.

The statistical significance of a result is the probability that the observed relationship (e.g., between variables) or a difference (e.g., between means) in a sample occurred by pure chance (“luck of the draw”), and that in the population from which the sample was drawn, no such relationship or differences exist. Using less technical terms, we could say that the statistical significance of a result tells us something about the degree to which the result is “true” (in the sense of being “representative of the population”).

More technically, the *P* value represents a decreasing index of the reliability of a result. The higher the *P* value, the less we can believe that the observed relation between variables in the sample is a reliable indicator of the relation between the respective variables in the population. Specifically, the *P* value represents the probability of error that is involved in accepting our observed result as valid, i.e., as “representative of the population.” For example, a *P* value of 0.05 (i.e., 1/20) indicates that there is a 5% probability that the relation between the variables found in our sample is a “fluke.” In other words, assuming that in the population there was no relation between those variables, whatsoever, and we were repeating experiments such as ours one after another, we could expect that approximately in every 20 replications of the experiment there would be one in which the relation between the variables in question would be equal or stronger than in ours. In many areas of research, the *P* value of 0.05 is customarily treated as a “cut-off” error level.

## HYPOTHESIS TESTING

In terms of selecting a statistical test, the most important question is “what is the main study hypothesis?”

In some cases there is no hypothesis; the investigator just wants to “see what is there”. For example, in a prevalence study, there is no hypothesis to test, and the size of the study is determined by how accurately the investigator wants to determine the prevalence. If there is no hypothesis, then there is no statistical test.

On the other hand, if a scientific question is to be examined by comparing two or more groups, one can perform a statistical test. For this, initially a null hypothesis needs to be formulated, which states that there is no difference between the two groups. It is expected that at the end of the study, the null hypothesis is either rejected or not rejected. It is equally essential to identify suitable test parameters to compare the groups.[1]

For example, in a clinical study to investigate whether an antihypertensive drug works better than placebo, the test variable can be the reduction in diastolic blood pressure (BP), calculated from the mean difference in BP between the active treatment and placebo groups. The null hypothesis is, “there is no difference between the active treatment and the placebo with respect to antihypertensive activity”.

A statistical test then calculates the probability of obtaining the observed difference between the two groups and tells us whether the observed difference is due to chance or real (statistically significant).[2,3]

A small *P* value means that this probability is slight. The null hypothesis is rejected if the *P* value is less than a level of significance which has been defined in advance. In our case, there might be the difference in mean BP after 6 months. If the value of the test variable is greater or lesser than a specific limit, it is unlikely that the null hypothesis is correct and the null hypothesis is accordingly rejected.[4] The result is then “statistically significant at the level α”.

## DECIDING A STATISTICAL TEST

The selection of the statistical test before the study begins ensures that the study results do not influence the test selection.

The decision for a statistical test is based on the scientific question to be answered, the data structure and the study design. Before the data are recorded and the statistical test is selected, the question to be answered and the null hypothesis must be formulated. The test and the level of significance must be specified in the study protocol before the study is performed. It must be decided whether the test should be one-tailed or two-tailed. If the test is two-tailed, this means that no particular direction of expected difference is assumed. One does not know whether there is a difference between the new drug and placebo with respect to efficacy. It is unclear in which direction the difference may be. (The new drug might even work less well than the placebo.) A one-tailed test should only be performed when there is clear evidence that the intervention should only act in one direction. The outcome variable (endpoint) is defined at the same time the question to be answered is formulated. Three criteria are decisive for the selection of the statistical test, which are as follows:

- the number of variables,
- types of data/level of measurement (continuous, binary, categorical) and
- the type of study design (paired or unpaired).

### The number of variables that the test is to be conducted on

Statistical tests and procedures can be divided according to the number of variables that they are designed to analyze. Therefore, when choosing a test it is important that you consider how many variables one wishes to analyze. One set of tests is used on single variables (often referred to as descriptive statistics), a second set is used to analyze the relationship between two variables and a third set used to model multivariable relationships (i.e., relationships between three or more variables).

### Types of data: Continuous, categorical, or binary

For example, in the comparison of two antihypertensive drugs, the endpoint can be the change in BP in the two treatment groups. The change in BP is a continuous endpoint. It is also necessary to distinguish whether a continuous endpoint is (approximately) normally distributed or not.

If, however, one only considers whether the diastolic BP falls under 90 mm Hg or not, the endpoint is then categorical. It is binary, as there are only two possibilities. If there is a meaningful sequence in the categorical endpoints, this can be described as an “ordinal endpoint ”, e.g., levels of service satisfaction range from highly dissatisfied to highly satisfied; also, attitude scores representing degree of satisfaction or confidence and preference rating scores.

### Paired and unpaired study designs

A statistical test is used to compare the results of the endpoint under different test conditions (such as treatments). There are often two therapies. If results can be obtained for each patient under all experimental conditions, the study design is paired (dependent). For example, two times of measurement may be compared, or the two groups may be paired with respect to other characteristics.

Typical examples of pairs are studies performed on one eye or on one arm of the same person. Typical paired designs include comparisons before and after treatment. For example “matched pairs” in case–control studies are a special case. These involve selecting a group where each subject is matched to a particular subject in the other group and they necessitate that the two groups be equal in size. The data are then no longer independent and should be treated as if they were paired observations from one group.[2]

With an unpaired or independent study design, results for each patient are only available under a single set of conditions. The results of two (or more) groups are then compared. The group sizes can be either equal or different.

## COMMON STATISTICAL TESTS

The most important statistical tests are listed in Table 1. A distinction is always made between “categorical or continuous” and “paired or unpaired.”

**Table 1:** Most important statistical tests

Statistical test	Description
Fisher’s exact test	Suitable for binary data in unpaired samples: The 2 × 2 table is used to compare treatment effects or the frequencies of side effects in two treatment groups
Chi-square test	Similar to Fisher’s exact test (albeit less precise). Can also compare more than two groups or more than two categories of the outcome variable. Preconditions: sample size ca. >60; expected number in each field 5
McNemar test	Preconditions similar to those for Fisher’s exact test, but for paired samples
Student’s t-test	Test for continuous data. Investigates whether the expected values for two groups are the same, assuming that the data are normally distributed. The test can be used for paired or unpaired groups
Analysis of variance	Test preconditions as for the unpaired t-test, for comparison of more than two groups. The methods of analysis of variance are also used to compare more than two paired groups
Wilcoxon’s rank sum test (also known as the unpaired Wilcoxon rank sum test or the Mann–Whitney U test)	Test for ordinal or continuous data. In contrast to Student’s t-test, does not require the data to be normally distributed. This test too can be used for paired or unpaired data
Kruskal–Wallis test	Test preconditions as for the unpaired Wilcoxon rank sum test for comparing more than two groups
Friedman test	Comparison of more than two paired samples, at least ordinal scaled data
Log rank test	Test of survival time analysis to compare two or more independent groups
Pearson correlation test	Tests whether two continuous normally distributed variables exhibit linear correlation
Spearman correlation test	Tests whether there is a monotonous relationship between two continuous, or at least ordinal, variables

### Tests used for group comparison of two categorical endpoints

**Figure 1:**
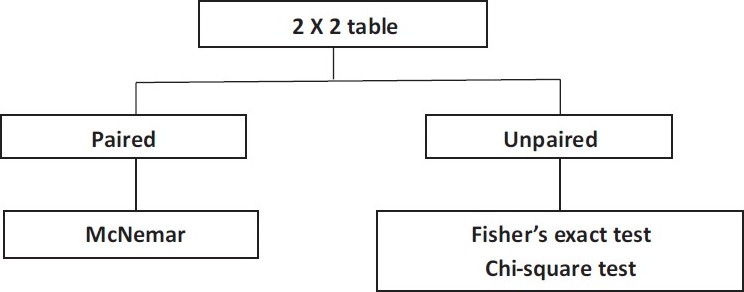
Test selection for group comparison with two categorical endpoints.

The group comparison for two categorical endpoints is illustrated here with the simplest case of a 2 × 2 table (four-field table) [Figure 1]. However, the procedure is similar for the group comparison of categorical endpoints with multiple values [Table 1].

#### Unpaired samples

If the frequency of success in two treatment groups is to be compared, Fisher’s exact test is the correct statistical test, particularly with small samples. For large samples (about *N* > 60), the chi-square test can also be used [Table 1].

#### Paired samples

One example of the use of this test would be an intervention within a group at two anatomical sites, such as the implantation of two different sorts of intraocular lenses (IOLs) in the right and left eyes, with the endpoint “Operation successful: Yes or no.” The samples to be compared are paired. In such a case, one has to perform the McNemar test. One more example for McNemar test is drug dose versus insomnia.

### Tests used for continuous and at least ordinally scaled variables

**Figure 2:**
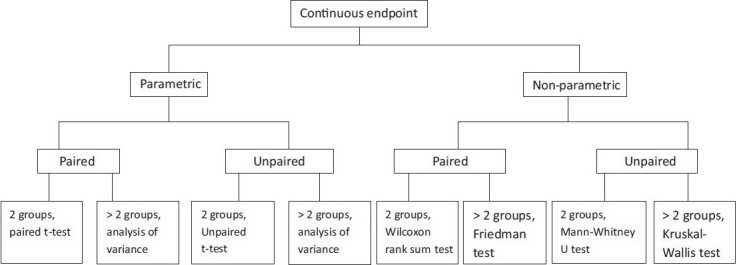
Algorithm for test selection for group comparison of a continuous endpoint.

Figure 2 shows a decision algorithm for test selection.

### Normally distributed variables–Parametric tests

The so-called parametric tests can be used if the endpoint is normally distributed.

#### Unpaired samples

Where subjects in both groups are independent of each other (persons in first group are different from those in second group), and the parameters are normally distributed and continuous, the unpaired *t*-test is used. If a comparison is to be made of a normally distributed continuous parameter in more than two independent (unpaired) groups, analysis of variance (ANOVA) can be used. One example would be a study with three or more treatment arms. ANOVA is a generalization of the unpaired *t*-test. ANOVA only informs whether the groups differ, but does not say which groups differ. This requires methods of multiple testing.[3]

#### Paired samples

The paired *t*-test is used for normally distributed continuous parameters in two paired groups. If a normally distributed continuous parameter is compared in more than two paired groups, methods based on ANOVA are also suitable. The factor describes the paired groups—e.g., more than two points of measurement in the use of a therapy.

### Non-normally distributed variables–Non-parametric tests

If the parameter of interest is not normally distributed, but at least ordinally scaled, nonparametric statistical tests are used. One of these tests (the “rank test”) is not directly based on the observed values, but on the resulting rank numbers. This necessitates putting the values in order of size and giving them a running number. The test variable is then calculated from these rank numbers. If the necessary preconditions are fulfilled, parametric tests are more powerful than non-parametric tests. However, the power of parametric tests may sink drastically if the conditions are not fulfilled.

#### Unpaired samples

The Mann–Whitney U test (also known as the Wilcoxon rank sum test) can be used for the comparison of a non-normally distributed, but at least ordinally scaled, parameter in two unpaired samples.[4] If more than two unpaired samples are to be compared, the Kruskal–Wallis test can be used as a generalization of the Mann–Whitney U test.[5]

#### Paired samples

The Wilcoxon signed rank test can be used for the comparison of two paired samples of non-normally distributed parameters, but on a scale that is at least ordinal.[5] Alternatively, the sign test should be used when the two values are only distinguished on a binary scale—e.g., improvement versus deterioration. If more than matched paired samples are being compared, the Friedman test can be used as a generalization of the sign test.

## CONCLUSIONS

Before selecting a statistical test, a researcher has to simply answer the following six questions, which will lead to correct choice of test.

1. How many independent variables covary (vary in the same time period) with the dependent variable?
2. At what level of measurement is the independent variable?
3. What is the level of measurement of the dependent variable?
4. Are the observations independent or dependent?
5. Do the comparisons involve populations to populations, a sample to a population, or are two or more samples compared?
6. Is the hypothesis being tested comparative or relationship?
